# Evaluation of Pattern Recognition Methods for Head Gesture-Based Interface of a Virtual Reality Helmet Equipped with a Single IMU Sensor

**DOI:** 10.3390/s19245408

**Published:** 2019-12-08

**Authors:** Tomasz Hachaj, Marcin Piekarczyk

**Affiliations:** Institute of Computer Science, Pedagogical University of Krakow, 2 Podchorazych Ave, 30-084 Krakow, Poland; marcin.piekarczyk@up.krakow.pl

**Keywords:** IMU sensors, pattern recognition, motion capture, head gestures, dynamic time warping, quaternions, principal components analysis

## Abstract

The motivation of this paper is to examine the effectiveness of state-of-the-art and newly proposed motion capture pattern recognition methods in the task of head gesture classifications. The head gestures are designed for a user interface that utilizes a virtual reality helmet equipped with an internal measurement unit (IMU) sensor that has 6-axis accelerometer and gyroscope. We will validate a classifier that uses Principal Components Analysis (PCA)-based features with various numbers of dimensions, a two-stage PCA-based method, a feedforward artificial neural network, and random forest. Moreover, we will also propose a Dynamic Time Warping (DTW) classifier trained with extension of DTW Barycenter Averaging (DBA) algorithm that utilizes quaternion averaging and a bagged variation of previous method (DTWb) that utilizes many DTW classifiers that perform voting. The evaluation has been performed on 975 head gesture recordings in seven classes acquired from 12 persons. The highest value of recognition rate in a leave-one-out test has been obtained for DTWb and it equals 0.975 (0.026 better than the best of state-of-the-art methods to which we have compared our approach). Among the most important applications of the proposed method is improving life quality for people who are disabled below the neck by supporting, for example, an assistive autonomous power chair with a head gesture interface or remote controlled interfaces in robotics.

## 1. Introduction

Virtual reality helmet (VRH) is a head-mounted device that allows a user to interact with a three-dimensional virtual reality environment by displaying stereovision images. VRH also analyses user’s head position and adjusts the virtual camera position in the virtual reality world in order to enhance the immersive experience.

Virtual reality systems might contain several types of user interface depending on the application of such solution. It can be either full-body motion capture (MoCap), both visual and sensor-based, hand gestures-based interface supported by various handled devices, gaze-based interface or head motion interface (also called head gestures interface). The last type of interface might be used without any additional handled devices and motion acquisition systems beside internal measurement unit (IMU) sensors in VRH. Gaze gestures are a promising input technology for wearable devices, especially in smart glasses form because gaze gesturing is unobtrusive and leaves the hands free for other tasks [1,2]. Gaze gestures utilize eye tracking technology and, to be reliable, it requires dedicated hardware. Alternatively, a system can track user head motion trajectory and analyze spatial motion patterns. This type of interface depends less on specialized cameras and can be developed using even IMU of smartphones. The contemporary IMU of smartphone devices has many applications. Among them is analysis and recognition of human activity—a survey on this subject can be found in in [3,4]. Beside IMU sensors, an up to date smartphone device contains efficient GPU and high resolution screen. Thanks to those features, smartphones can be used to construct a relatively affordable component of virtual reality glasses/helmets [5]. Head gestures require a pattern recognition algorithm that would enable unambiguous recognition of user head trajectory. In contrast to other types of human activity recognition this problem has not been investigated as much as, for example, gait or hand motions classification and analysis. In the following section we will present an up-to-date summary of the most effective methods for head motion analysis and for similar human motion evaluation tasks.

### 1.1. Head Motion Analysis and Classification

Surprisingly, to our best knowledge there are only a few papers that directly address the problem of head gestures of application for user interface or who discuss the broader subject of head gestures recognition. Some of them present a very initial study and in some cases, a lack of available data and detailed description of applied methods makes the presented results impossible to reproduce. In [6] the authors have applied Dynamic Time Warping (DTW) algorithm in order to calculate the similarity between time sequences acquired with IMU sensors in order to classify four classes of actions. Application of weighted DTW classifier for head motions acquired by Smart Glasses is reported in [7]. However, due to a lack of detailed description of methodology and data it is hardly possible to reproduce the method and results. In [8,9], Hidden Markov Models (HMM) are used for head motion classification to support sign gestures recognition and recognition of four types of actions respectively. Those two papers also do not use publicly available datasets. In summary, methods that are commonly applied in head gestures classification works on unprocessed time series, which means that those classifiers take as an input data a multivariate time series that is segmented from an incoming data stream. Especially popular is DTW time series classification algorithm which utilizes the similarity function to determine the similarity of the time series pairs [10,11]. Head gestures recognition, however, is a special case of other body parts motion recognition or more generally human motions or gait recognition. There are no contradictions in applying the most effective methods of those types to head trajectory classification. We will discuss this in the following section.

### 1.2. Effective Methods of Human Motion Analysis and Classification

While head motion in VRH is often tracked using a single IMU sensor, the produced time series involve either a three or four dimensional signal depending of type of motion description that is applied. It is three dimensional time series if the data is represented as rotation using Euler angles or if it represented as coordinates of normalized vectors that indicate head orientation (colloquially speaking, the direction of those vectors shows direction of the user’s nose). It is a four dimensional time series if rotation is represented as quaternions. Due to this fact, some methods present in literature also utilize three or four dimensional time series with motion descriptions (as rotation or vector direction) that can also be applied for head gestures recognition. Those methods (for example, for hand motion classification) utilize various classifiers depending on motions features selection, for example DTW, HMM or when features are derived after processing original motion capture data nearest neighbor classifier (NN), support vector machine (SVM) or neural network [12,13]. A good example of this is [14] where the authors apply HMM to classify multidimensional hand motion data composed of Euler angles rotation, gyroscope and accelerometer data. An algorithm from [15] uses the Naive-Bayes-nearest-neighbor classifier to measure distance between joint-based histogram representation of full body motion and the action to be classified.

Analysis of full body MoCap is a more general problem, because it utilizes several dozen body joints measurements. Those tasks require methodologies that enable scaling influence (importance) of separate joints on overall data recognition. Among the methods that are becoming more and more popular in the last years are neural network (ANN) and deep neural network (DNN)-based methods. If action recognition is performed on raw video data authors prefer to use convolution neural networks where convolution layer is used to generate features. Those features are then processed by a fully connected neural network which performs classification [16]. Sometimes an input raw signal is processed by convolution layer followed by recurrent network to avoid a sliding window design and then classified by a fully connected neural network [17]. In situations when appropriate motions features are already present an ANN can be applied directly [18,19]. In some research, authors also utilize DTW [20]. However, as can be seen in papers that contain a comprehensive survey on this subject [12,13,21,22] Principal Components Analysis (PCA) is a commonly used procedure for dimensionality reduction and features set generation for human motions analysis and classification. Because application of PCA in various steps of motion analysis and classification seems to be a standard approach for gait/full body motion analysis (although it was not mentioned in state-of-the-art papers devoted to head gestures recognition) the rest of this survey will be devoted to PCA.

Generally, PCA-based time series processing is used either for dimensionality reduction before a signal is classified by a pattern recognition algorithm or another type of analyses. This is done because in many cases distance-based classifiers perform better in lower dimensional space. The choice of following pattern recognition method or analysis procedure depends on what the goal authors wanted to obtain. In the case of classification, authors use, for example, HMM [23], DTW [24], nearest neighbors [25,26,27], and SVM [28]. Authors also perform various types of analysis for example aligning signals with Two Step Aligning algorithm [29], drawing direct conclusions from principal components [30,31,32,33] or data segmentation [34]. The second application of PCA is for generating motion features of fixed size that are vector of real values which represents features of linear combination of eigenvectors that can be also classified by general-purpose classifiers like, for example, SVM [35,36,37] or nearest neighbors [38]. The classification effectiveness depends mostly on type of activity authors wanted to recognize the quality and variability of data. There are also some methods that utilize both dimensionality reduction and Eigen-based features to perform motion classification, for example, [39].

### 1.3. Motivations of This Paper

As can be seen in previous section head gestures classification and its application for virtual reality interfaces have not been a target of extensive research yet. This might be because of the fact that VRG based on smartphone hardware (IMU, GPU and screen) has become widely available in last few years and there was not much interest in this type of pattern recognition solution before. MoCap technology was either used for whole body (gait/posture recognition and analysis) by general purpose MoCap systems or a dedicated IMU-based hardwares were constructed for certain tasks (see for example [6]). The motivation of this paper is to fill that gap by examining the effectiveness of state-of-the-art and newly proposed MoCap pattern recognition methods on the task of head gestures classifications. We will validate a classifier based on PCA features with various numbers of dimensions for the two-stage PCA recognition method [39]. In [18], the author performs classification of a high-frequency EMG seven-dimensional signal using several pattern recognition techniques among them artificial neural network (ANN) and decision trees (DT). We adapted methods from that paper to our pattern recognition tasks. We have replace however DT with a Random Forest (RF) classifier to prevent a single DT overfitting to training dataset. In this paper we introduce DTW classifier trained with extension of DBA algorithm that utilizes quaternion averaging [40] and we will also proposes a bagged variation of previous method that utilizes many DTW classifiers that perform voting to obtain final classification. The last classifier and its training procedure based on [40] is our original contribution. Both an original data set that was used for this evaluation, Android OS software for data acquisition and all R-language source codes we used to conduct the research is available to download in order to make our research reproducible and to check methodology and implementation details (The source codes and data can be download from https://github.com/browarsoftware/headmotions). Some methods that we are comparing with our approach, although they were developed several years ago, are extensively used in up-to-date researches like for example DTW-based classification [14] or various application of PCA-based dimensionality reduction and features selection [21,32]. Also, SVM and KNN classifiers [15] are often utilized to classify data acquired by IMU sensors. The most important difference between all those papers is that depending on the motion acquisition technique, authors need to perform various preprocessing of the MoCap signal to make it applicable to dimensionality reduction and patter recognition techniques. Those methods are specific to signal domain (for example raw video data, EMG, full body MoCap, so-called skeleton) and are not within the scope of this paper. As we previously mentioned in Section 1.1, there are not many up to date papers devoted to head gestures so we evaluate the proposed approaches with methods that were successful in other motion types.

A very important factor that was not taken into account in previous research on head motion analysis is supporting a user who performs the head motion with some visual feedback: an actual trajectory of his/her head. Such type of feedback (for example drawing points that indicate actual head direction in virtual or augmented reality technology) might highly increase the awareness of motion trajectories that are drawn by a user’s head. The dataset we used in this research was gathered using a VRH with systems that draw such a trajectory. This is why we used whole VRH rather than only an IMU sensor.

Methods presented in this paper have many important applications. Among the most important are the possibility of improving life quality for disabled people below the neck for example by supporting assistive autonomous powerchair with head gestures interface (see [6]) or remote controlling interfaces in robotics [41,42]. Technologies that utilize head-mounted devices have been mentioned in several researches in the last years. Among its possible applications is augmented reality technology for pedestrian collision warning [43], driver drowsiness detection systems [44], eye tracking-based interfaces [45], and drivers’ distraction detection [46,47]. Especially the last type of application might be a promising field of application for our algorithm because some head positions and motions might be an indicator that a driver is sleepy or falls asleep. We have to remember, however, that while using IMU sensors in high speed vehicles like cars, we should utilize an additional method to distinguish the motion of the vehicle and the device’s motion in the vehicle [48] and also compensate for vehicle shaking [49].

## 2. Materials and Methods

### 2.1. Evaluation Dataset

Because we have not found a publicly available dataset that contains head MoCap data acquired by smartphone-based VHR the dedicated dataset has been created. The data we gathered is saved as a three-dimensional stream of Euler angles rotations of head and four-dimensional stream of quaternions that represents the same data. Let us recall some elements of quaternion’s algebra that are necessary to define the methods we have used. Let us assume that we have two quaternions: $q_{1}=\left[x_{1},y_{1},z_{1},w_{1}\right]$, $q_{2}=\left[x_{2},y_{2},z_{2},w_{2}\right]$ where *x*, *y*, *z* and *w* are quaternion coordinates.

Quaternion normalization:

(1) $$U_{q_{1}}=\frac{q_{1}}{\sqrt{x_{1}^{2}+y_{1}^{2}+z_{1}^{2}+w_{1}^{2}}}$$

Multiplication of quaternions $q_{1}$ and $q_{2}$:

(2) $$q_{1}\cdot q_{2}=\left[\begin{array}{c} w_{2}\cdot x_{1}+x_{2}\cdot w_{1}+y_{2}\cdot z_{1}-z_{2}\cdot y_{1},\\ w_{2}\cdot y1+y_{2}\cdot w_{1}+z_{2}\cdot x_{1}-x_{2}\cdot z_{1},\\ w_{2}\cdot z_{1}+z_{2}\cdot w_{1}+x_{2}\cdot y_{1}-y_{2}\cdot x_{1},\\ w_{2}\cdot w_{1}-x_{2}\cdot x_{1}-y_{2}\cdot *y_{1}-z_{2}\cdot z_{1} \end{array}\right]$$

Inverse of quaternion $q_{1}$:

(3) $$\begin{array}{c} q_{1}^{-1}=\frac{\left[-x_{1},-y_{1},-z_{1},w_{1}\right]}{d}\\ d=\sqrt{x_{1}^{2}+y_{1}^{2}+z_{1}^{2}+w_{1}^{2}} \end{array}$$

Angle between quaternions $q_{1}$ and $q_{2}$:

(4) $$\begin{array}{c} q_{3}=U_{q_{1}}\cdot U{q_{2}}^{-1}\\ \alpha =2\cdot acos(w_{3})\\ ∡\left(q_{1},q_{2}\right)=\left\{\begin{array}{ccc} \alpha & if & \alpha ⩽\pi \\ -\alpha & if & \alpha >\pi \end{array}\right. \end{array}$$

where $U{q_{2}}^{-1}$ is an inverse of normalized quaternion $q_{2}$ and $w_{3}$ is a *w* component of quaternion $q_{3}$.

**Figure 1:**
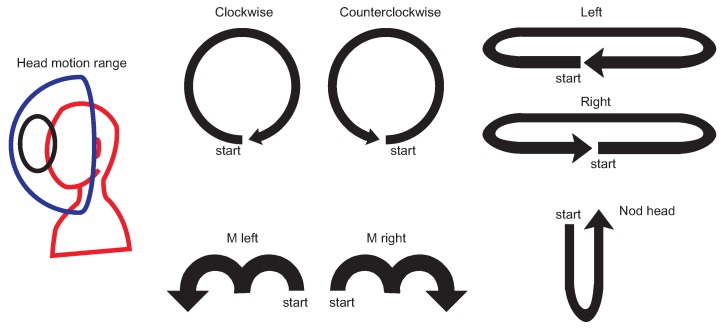
The left side of the figure presents schematic image of head motion range (a blue hemisphere) with example circular head gesture trajectory (black line). The right side of the figure presents “idealized” shape of motions in the evaluation dataset.

The evaluation dataset consisted of motions performed by twelve persons: eight males and four females whose ages varied from 15 to 61. All those persons were asked to perform seven types of head gestures at least ten times each; however, some of them performed many more repetitions. The dataset contains seven types of head gestures: clockwise rotation, counterclockwise rotation, left horizontal motion, right horizontal motion, head nodding, m-shaped move to left and m-shaped move to right. The “idealized” shape of motions are visualized in Figure 1. We have chosen those templates basing on reports from other papers, where authors have found them to be natural, especially for commands in design gestures-based controlling interface [6,7,50]. We have chosen this set of gestures for several reasons. All of them can be easily explained to and repeated by system users/experiment participants. Also, although there is a visible difference between each gesture, they have some important similarities, for example. circular trajectory is present both in clockwise and m-shaped actions. Also, actions have to be considered as the time-varying signals, because the spatial patterns that are drawn in space between some classes are undistinguishable when temporal information is not used. This situation happens, for example, for clockwise and counterclockwise, left and right, m left and m right actions pairs. The motion range of the head which is defined as a set of all possible normalized vectors coordinates that indicate head orientation has a semi hemispherical shape (see Figure 1, left). It is of course not possible to perform head motions that are 360 degrees rotation in axial (horizontal) plane; however, it is possible to make circular or m-shaped trajectories on the hemisphere of the motion range. The dataset we used is summarized in Table 1. We have acquired 975 motion recordings. MoCap data acquisition was performed every 22.06 +/− 5.83 ms. As can be seen there is large standard deviation in acquisition time that happens in each recording. That is because of the fact that a data gathering application does not have any mechanism that assures constant acquisition frequency. We have collected head gesture data using the hardware system based on smartphone Samsung Galaxy S7 (SGS7) and virtual reality headset equipment namely Google Cardboard. The phone has been powered by the Exynos 8890 with a Quad-Core 2.3 GHz Mongoose + Quad-Core 1.6 GHz Cortex A53 configuration and with memory LPDDR4 RAM of 4GB in size. The device operated under Android OS in version 8.0.

**Figure 2:**
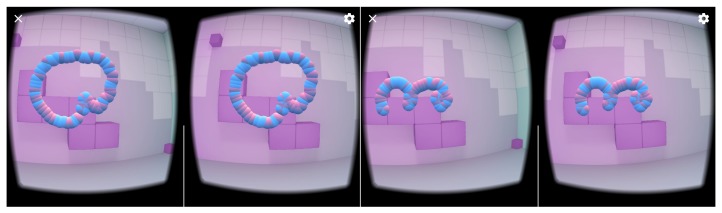
This figure presents stereovision view from the virtual reality helmet (VRH) system. The trajectory on the left is counterclockwise motion while the trajectory on the right is m right gesture.

**Figure 3:**
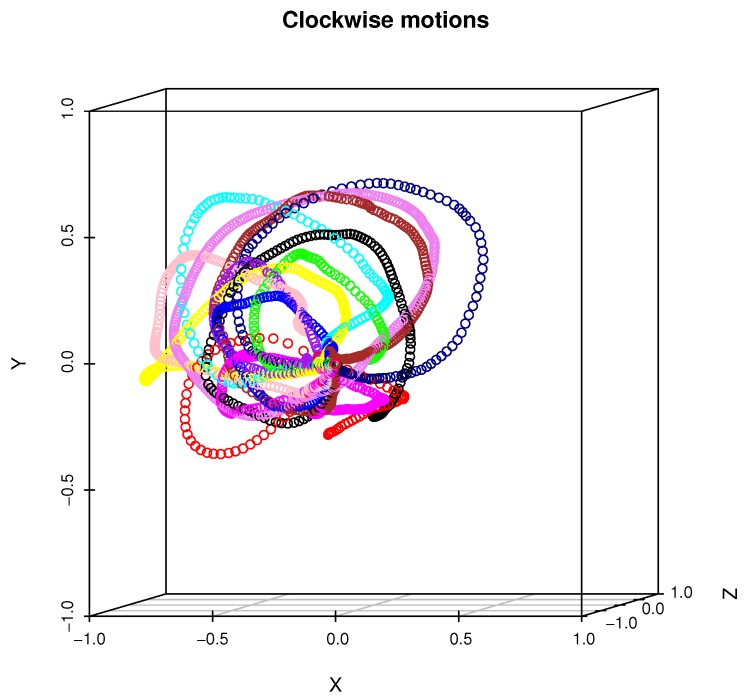
This figure presents twelve examples of a clockwise motion performed by each person. A trajectory that belongs to the single person is color-coded.

**Figure 4:**
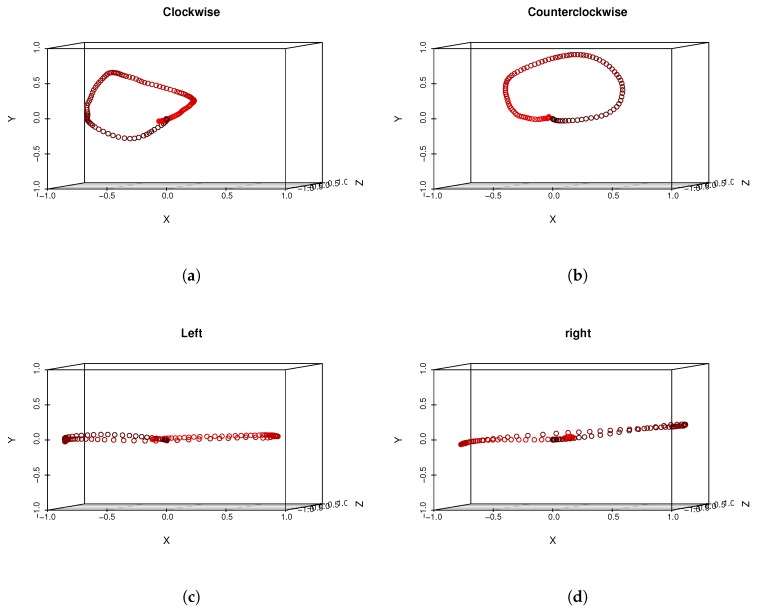

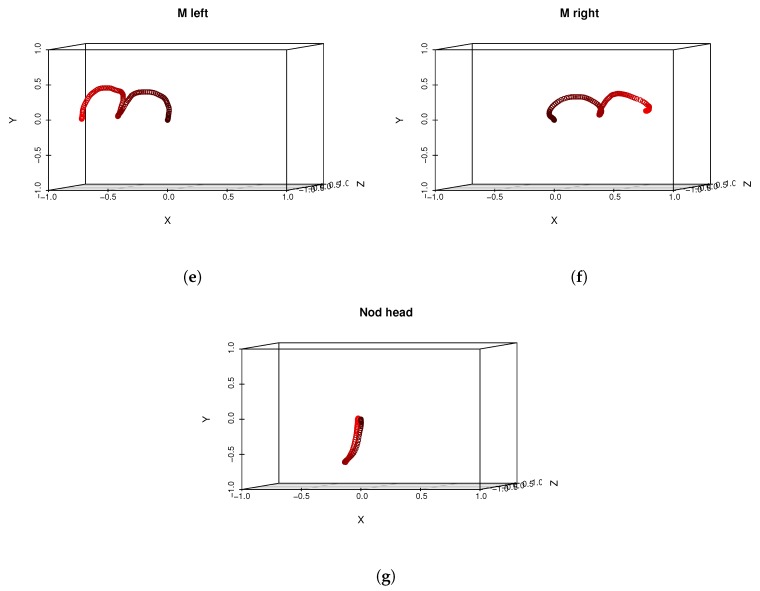
This figure presents real head motion trajectories obtained by one of the users – compare with Figure 1. Sampled head positions are color-coded. Early samples are darker while end samples of motions are red.

The phone of this model is equipped with an LSM6DS3 sensor manufactured by ST MICROELECTRONICS [51] with the full IMU functionality of 6-axis accelerometer and gyroscope. From technical point of view the LSM6DS3 sensor is a system-in-package featuring a 3D digital accelerometer and a 3D digital gyroscope performing at 1.25 mA (up to 1.6 kHz ODR) and able to work in different modes with linear acceleration measurement range of ±2g/±4g/±8g/±16g and angular rate measurement range of ±125dps/±245dps/±500dps/±1000dps/±2000dps appropriately. The data was acquired using an Android OS JAVA application developed by us, which visualizes the head motion trajectory of the user wearing VHR in the form of colored balls that were displayed in a virtual three-dimensional room, see Figure 2, for example. This additional visual feedback helped experiment participants to visualize themselves and which head motion they performed. As can be seen in Figure 3, each experiment participant might perform the same action in many ways: although motions are similar to idealized trajectory the obtained spatial representations of motions are very diverse. Figure 4 presents real head motion trajectories obtained by one of the users. Compare this with Figure 1.

**Figure 5:**
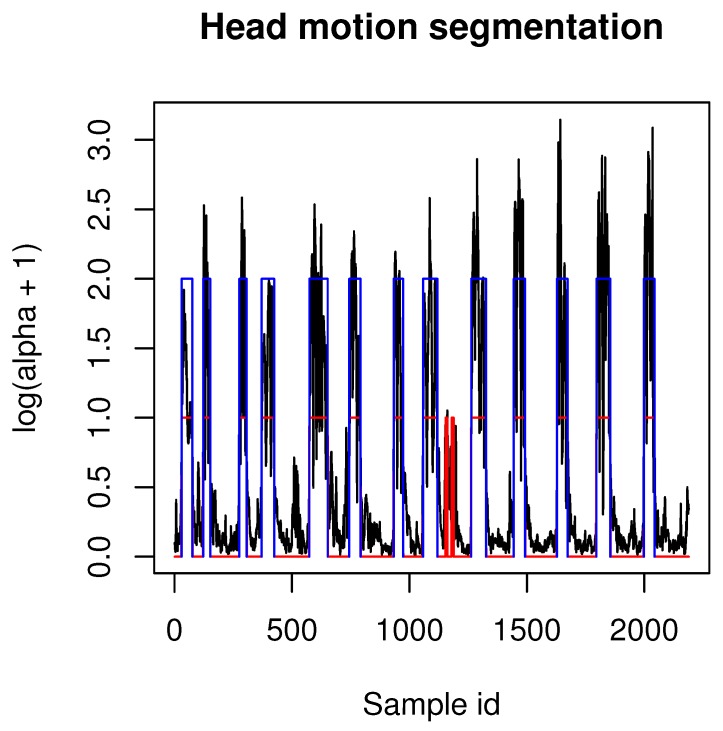
This figure presents example segmentation results of Algorithm 1. Black lines are angles between two neighboring quaternions (motion velocity), red segments are values above $t_{1}$ threshold of median-filtered velocity signal, blue segments are values above $t_{2}$ threshold. Those blue segments are segmentation results returned in list R.

The majority of recordings that were acquired by our application contained multiple actions performed one after another by experiment participants. In order to segment motions recordings into samples that contain only one example of a certain gesture (as it is present in Figure 4 and summarized in Table 1) we have used a heuristic segmentation algorithm presented in pseudocode Algorithm 1. It can be seen that the algorithm is dependent on three adaptive parameters: median filter window size, angle difference threshold (in degrees) and minimal length threshold. Values of those samples might differ depending on the MoCap system acquisition frequency and in our case we used values: mk = 15, $t_{1}=1$, $t_{2}=10$. After applying our algorithm to those values, all motions samples were correctly segmented from our MoCap data. After segmentation head gestures were manually labeled in order to assign them to correct classes. Example segmentation results are presented in Figure 5. We decided to use our own segmentation method because it have returned anticipated results; however, one can use any method proposed in the literature, for example, [34]; however, some tuning of those methods (for example, adaptation to acquisition frequency) might be unavoidable. Those issues, however, are beyond the scope of this paper.
**Algorithm 1:** Head gestures segmentation from quaternion signal
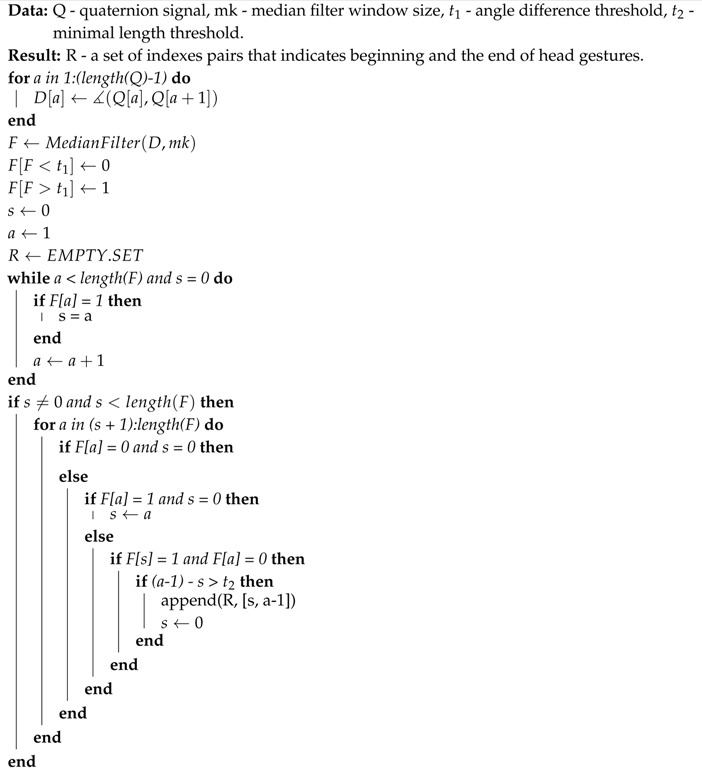


Each recording has been also rotated so that the initial rotation Euler angle has become [0, 0, 0] (that means [0, 0, 0, 1] for quaternion). In order to do so we have generated new rotation signal $Q_{r}$ from input rotation signal *Q* by multiplying the inverse quaternion of the first acquired frame $Q{\left[1\right]}^{-1}$ by each acquired frame Q[a]. After this operation, first the rotation equals [0, 0, 0, 1] and then the rest of them are transformed by the inverse of the initial rotation (see Equation (5)).

(5) $$\begin{array}{c} Q_{r}\left[a\right]=Q{\left[1\right]}^{-1}\cdot Q\left[a\right] \end{array}$$

In next sections we will describe the motion classification procedures we have tested in this research. The dataset preprocessed by methods described in this section is used by all the following algorithms.

### 2.2. Head Gestures Recognition with DTW Classifier

The first classification method we used is a DTW consisting of seven templates generated with modified DBA algorithm originally proposed in paper [40]. That method is a modification of DBA from [52]; however, it is adapted to operate on a quaternion signal. The original barycenter averaging method has been replaced by norm-preserving Markley’s algorithm [53] where the averaged quaternion is found by solving the following eigenvalue problem (see Algorithm 2).
**Algorithm 2:** Markley’s quaternion averaging
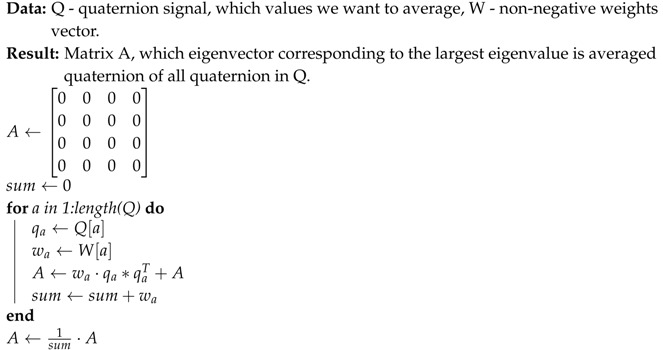

where ∗ is matrix (not quaternion!) multiplication operator.

The DTW cost function is defined as [20]:

(6) $$\begin{array}{c} cost\left(q_{1},q_{2}\right)=1-\left|q_{1}\circ q_{2}\right| \end{array}$$

where $\left|q_{1}\circ q_{2}\right|$ is an absolute value of a dot (scalar) product of quaternions $q_{1}$ and $q_{2}$.

The DTW distance is calculated as normalized DTW distance.

Our DTW classifier operates on spatial representation of head motion which is calculated using Equation (7). We can express head position in three-dimensional space as a unit vector [0,0,1] transformed by a quaternion that represents head rotation. Each head motion is represented as a three-dimensional time varying signal of spatial coordinates that denote normalized vectors that indicate head orientation. The coordinates of that vector drawn in three-dimensional space nicely represent head motion trajectory.

(7) $$\begin{array}{c} q_{1}^{*}=\left[-x_{1},-y_{1},-z_{1},w_{1}\right]\\ q_{v}=\left[0,0,1,0\right]\\ q_{r}=\left(q^{*}\cdot q_{v}\right)\cdot q_{1}\\ v_{1}=\left[x_{r},y_{r},zr\right] \end{array}$$

where $q_{1}$ is a rotation quaternion and $v_{1}$ is a return vector from that calculation.

The DTW classifier utilizes Euclidean distance and assigns input motion to the class for which the normalized distance between input signal and template signal that represents the motion class (also recalculated using Equation (7)) has the minimal value.

### 2.3. Bagged DTW Classier

The approach presented in Section 2.2 does not exploit the information from the training dataset about which the user performed a certain action. That is because, in the method presented in Section 2.2, each DTW template is generated from all MoCap recordings from training dataset. In order to apply a well-known bagging schema [54] to generate classifier from our dataset we can construct a set of “weak classifiers” where each of them is trained on data that come from the single user. This approach will generate as many DTW classifiers trained on motion classes as we have different experiment participants in our training dataset. Thanks to this approach, user-specific motions trajectories will not be averaged over all other trajectories. In the motion classification process, the set of “weak classifiers” performs voting and assigns input motion to the class to which motion was assigned by the majority of “weak classifiers”.

### 2.4. Head Gestures Recognition with PCA-Based Features

As we already mentioned in Section 1.2 PCA-based features are among most popular approaches used in MoCap data analysis and classification. That approach is described for example in paper [55] so we will summarize only the basic concept.

#### 2.4.1. Features Generation (Training)

At first, input data is processed in the same manner as in Section 2.1, also Equation (7) is used to recalculate quaternion representation to obtain spatial representation of head motion. Then we have to resample training data to the uniform size: each recording should have a common length. Also, all recordings that we would like to classify in the future will have to be resampled to that length. In the next step we perform the operation analogical to “flattening” known from deep neural networks. In this step we generate a single-dimensional vector from n-column input signal by putting values from each column one after another in a single vector. Suppose we have m rows and n columns. The flattened vector will have n·m rows and 1 column. Then we compose a matrix A, which columns are “flattened” vectors of training dataset. We create a mean vector $V^{\left[n\cdot m\right]}$ (in square brackets we present dimensionality of a vector, this is a column vector) whose values are calculated as averaged values of each row of matrix A. We generate a new matrix $A^{\prime }$ by subtracting $V^{\left[n\cdot m\right]}$ from each column of A. In the next step we calculate covariance matrix C from matrix A’ and we find eigenvalues and eigenvectors of C. We order eigenvalues by descending order and we take k eigenvectors that correspond with most significant eigenvalues. The PCA-based features that are used to describe a MoCap recording are coefficients of a linear combination of those most significant eigenvectors (or simply: vector coordinates) that are obtained after performing projection of input n·m-dimensional MoCap on k-dimensional space generated by Principal Components Analysis. That projection of an input signal $S^{\left[n\cdot m\right]}$ can be computed using the mean vector and a matrix composed of k eigenvectors corresponding to k most significant eigenvalues:

(8) $$S^{\left[k\right]}=X^{\left[k,n\cdot m\right]}\cdot \left(S^{\left[n\cdot m\right]}-V^{\left[n\cdot m\right]}\right)$$

where $X^{\left[k,n\cdot m\right]}$ is a matrix which rows are k eigenvectors corresponding to the k most significant eigenvalues.

We use the training dataset recalculated to be k-dimensional to train any desired classifier. In this paper we will utilize a nearest neighbor classifier.

#### 2.4.2. Classification

The classification step requires resampling of the recording we want to classify to the same length as it was done for a training dataset, flattening the input signal that represents motion and computing projection of obtained n·m vector to k-dimensional space using coefficients calculated in feature generation (training) step (8). Then we classify the input recording using appropriate classifier (as we already mentioned in our case it is a nearest neighbor classifier). In our implementation we have used unoptimized NN algorithm, where we compare input vector to each other vector in the dataset to find the nearest neighbor. We are aware of the existence of searching algorithms that accelerates computation like kd-trees [56] Semi-Convex Hull Tree [57] or Dynamic Continuous Indexin [58]; however, they will not improve the recognition rate of the approach, which was a goal of our research.

### 2.5. Two-Tage PCA-Based Method

In some approaches, the authors perform an additional initial projection of input data to lower dimensional space with PCA before calculating PCA-based features. For example, in [39], the authors project whole dataset to two-dimensional space with PCA, then they calculate PCA-based features using approach presented in Section 1. The classification is performed using SVM classifier. Input data for the algorithm is processed in the same manner as in Section 2.1, also Equation (7) is used to recalculate quaternion representation to obtain spatial representation of head motion.

### 2.6. Neural Network and Random Forest

Similarly to [18] we have applied ANN (a feedforward artificial neural network) and DT-based approaches (namely RF) to perform classification of our data. Dimensionality reduction is a commonly-used procedure that is applied on a high-dimensional dataset or signals with a large number of samples. However, linear projection might be redundant while the classification process is done with a classifier capable of partitioning the features space with appropriate decision hyperplanes. Also, in our case it is not necessary to apply convolution layers to calculate input features from time series, because the coordinates of vectors that represent head motion trajectory (7) are enough to train a fully connected network or a decision tree. In our case the only requirement for the training dataset is to perform resampling to the uniform size and “flattening”, as described in Section 2.4.1. That resampled and “flattened” signal is used as an input data for NN and RF algorithms. The NN activation function was hyperbolic tangent [18] and the network was trained using s parallelized version of stochastic gradient descent using back-propagation [59]. We have implemented these machine learning methods using the H2O library (https://github.com/h2oai/h2o-3).

## 3. Results

**Figure 6:**
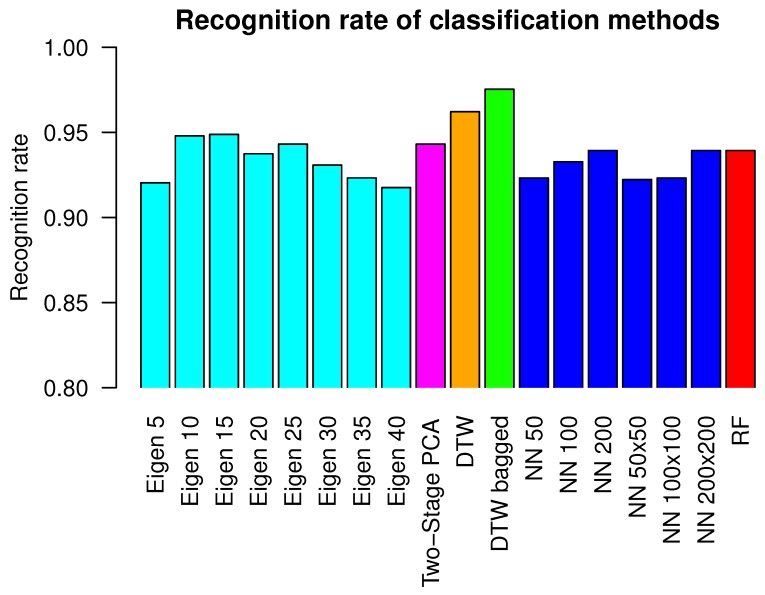
This figure visualizes results from Table 2 in the form of bar plot.

We have evaluated the methods presented in Section 2.2, Section 2.3, Section 2.4 and Section 2.5 on the dataset from Section 2.1. All classifiers have been evaluated using leave-one-out cross validation method, where recordings from the single person have been used for testing while recordings from eleven persons have been used for classifier training. In case of the NN classier with PCA-based features from Section 2.4, we have evaluated various lengths of features vectors: with 5, 10, 15, 20, 25, 30, 35 and 40 coefficients. Because highest recognition for that method was obtained for a 15-dimensional vector, we have used the same length of vector in the second step of Two-stage PCA-based method from Section 2.5. Several ANN architectures have been tested with a different number of neurons in the hidden layer. We have used a hidden layer with a single row of 50, 100 or 200 neurons and a hidden layer with two, fully connected rows of neurons, each of them with size 50, 100 or 200. Random forest contained 50 decision trees with maximal depth 20. In the case of Bagged DTW classifiers, they contained eleven “weak classifiers”, each of them trained on data from a different person. In Table 2 we present the error rate of each examined classifier and total error rate of each classifier is presented. The recognition rate is defined as number of correctly classified objects of that class divided by all objects of that class, the error rate equals 1− recognition rate. Total recognition rate is defined as number of correctly classified objects of each class divided by all objects of all classes. Total error rate is 1− total recognition rate. Figure 6 visualizes the results from Table 2 in the form of a bar plot. The highest value of recognition rate has been obtained for hte DTW bagged classifier. It equals 0.975 and is higher than the second-best classifier, namely DTW, by 0.013. DTW bagged error rate is the smallest or it shares smallest position in case of clockwise, counterclockwise, left and right head gestures. The multiclass confusion matrix for the DTW bagged classifier is presented in Table 3. Table 4 presents the same confusion matrix, however each value is calculated relatively to the count of head gestures of each type. Table 4 contains an additional column that contains the classification error for each motion class.

## 4. Discussion

As can be seen in the previous section, all methods that were applied for head gestures classifications have obtained very good results on our test dataset. Each classifier that was applied for this task obtained total recognition rate above 91%. Figure 3 visualizes why the classification was not an easy task: motions of each person varied in execution speed, range of head rotation and spatial head position. The reason for such a high recognition rate the a fact that, for this evaluation, we have chosen pattern recognition methods that have either already proven their usefulness in MoCap data identification, or we have anticipated them to be very promising. Error rates obtained by various architectures of ANN and a RF are on a similar level as error rates of the NN algorithm with 25–35 PCA features. This might be explained by the fact that ANN and RF utilize decision hyperplanes that might substitute dimensionality reduction performed by PCA. We have observed the lowest error rates for ANN with a single hidden layer of 100 neurons and two rows of 50 neurons each. In case of networks that contained less or more neurons or layers, results were worse. That might be caused by classifiers’ underfitting or overfitting appropriately. Two methods that utilized DTW with template signals averaged by algorithm [40] have a higher recognition rate than performing methods that utilized various numbers of PCA-based features. The most probable reason for this is the fact that PCA-based features deal very well with MoCap data that contain signals from several body joints because they have the ability to linearly map the initial signal space to lower-dimensional space that maximizes the variance between recordings. In the case of motions gathered from the single sensors, however, it is possible to obtain higher recognition rate using direct matching of motions trajectories to averaged signal using DTW. Also, the DTW method does not require input signals resampling to the uniform length. The bagged version of the DTW classifier has slightly higher recognition rate than the non-bagged version. The bagged classifier has higher computational complexity than non-bagged; however, classification in each "weak" classifier is performed independently and can be paralleled without much influence on overall classification time. The evaluation proved that in the case of all classifiers, there were some recognition errors. Because of the leave-one-out procedure, the training dataset did not contain data acquired from a person on whose data validation was done. In all validations, there was no event when all test data was misclassified; however, there were situations when not all recordings in the validation dataset were assigned to the correct class. That means that all applied classifiers were biased due to variability, which happens during several repetitions of same action by people in the dataset.

## 5. Conclusions

In this paper, we propose and evaluate several methods capable of classifying head gestures acquired by VRH powered by of-the-shelf smartphone hardware. We have tested all methods on a large dataset of head motions for which trajectories were recommended as the benchmark motions for gesture-based interfaces. The results we have obtained proved that the bagged DTW classifier trained with modified DBA algorithm that averages quaternion-based signal is the most promising algorithm for that task. DTW also has an important advantage over methods that utilized PCA-based features because DTW does not require input signal resampling to the uniform length. Due to this fact, DTW can directly compare the input signal to the templates after segmentation and processing with (5). As we already mentioned in the first section of this paper the most important application of our method is head motion-based interface for disabled people. The results we have obtained are not only limited to head gestures acquired with VRH with smartphone hardware but can be generalized to any MoCap method that allow the measuring of head motion. In future work, we plan to extend our research on eye tracking technology and eye motions and gaze-based interfaces. This is also a very challenging subject because eyes perform both voluntary and involuntary movements and we anticipate that the main obstacle to overcome is to distinguish one type of these motions from another.

## Figures and Tables

**Table 1:** This table summarizes data in evaluation dataset. Rows represents persons while columns represents motion classes. MIN and MAX are minimal and maximal number of samples for each motion class that are present in the recordings. For example, the shortest clockwise recording has 85 samples while the longest has 332 samples.

	Clockwise	Counterclockwise	Left	Nod Head	M Left	M Right	Right
1	10	11	11	12	12	12	12
2	12	13	11	13	11	11	11
3	14	11	11	11	11	11	11
4	10	10	11	11	13	13	11
5	12	11	12	23	11	11	11
6	11	11	12	11	12	11	11
7	11	12	10	12	11	10	10
8	11	12	11	13	11	12	11
9	11	12	11	12	12	12	11
10	12	11	11	22	12	12	11
11	11	12	11	11	11	11	11
12	14	11	11	10	10	11	10
SUM	139	137	133	161	137	137	131
MIN	85	86	81	50	75	76	81
MAX	332	412	528	183	255	264	406

**Table 2:** This table presents error rate of each examined classifier and total error rate of each classifier.

	Clockwise	Counterclockwise	Left	Nod Head	M Left	M Right	Right	Error
Eigen 5	0.148	0.074	0.090	0.035	0.054	0.020	0.147	0.080
Eigen 10	0.101	0.074	0.030	0.075	0.007	0.027	0.049	0.052
Eigen 15	0.054	0.095	0.028	0.087	0.020	0.020	0.049	0.051
Eigen 20	0.074	0.115	0.014	0.110	0.020	0.074	0.021	0.063
Eigen 25	0.067	0.088	0.028	0.104	0.013	0.047	0.042	0.057
Eigen 30	0.081	0.095	0.042	0.098	0.034	0.081	0.049	0.069
Eigen 35	0.074	0.115	0.042	0.098	0.067	0.081	0.056	0.077
Eigen 40	0.087	0.122	0.042	0.110	0.094	0.074	0.042	0.082
Two-Stage PCA	0.134	0.061	0.069	0.046	0.034	0.040	0.014	0.057
DTW	0.087	0.014	0.000	0.029	0.054	0.067	0.014	0.038
DTW bagged	0.020	0.007	0.000	0.040	0.027	0.060	0.014	0.025
NN 50	0.087	0.115	0.035	0.150	0.013	0.013	0.112	0.077
NN 100	0.094	0.088	0.028	0.127	0.027	0.013	0.084	0.067
NN 200	0.087	0.115	0.035	0.069	0.020	0.007	0.091	0.061
NN 50x50	0.081	0.122	0.028	0.087	0.020	0.020	0.063	0.061
NN 100x100	0.114	0.095	0.028	0.150	0.020	0.007	0.112	0.077
NN 200x200	0.107	0.101	0.035	0.168	0.027	0.007	0.084	0.078
RF	0.081	0.101	0.021	0.104	0.027	0.007	0.077	0.061

**Table 3:** This table presents the multiclass confusion matrix for DTW bagged classifier.

	Clockwise	Counterclockwise	Left	Nod Head	M Left	M Right	Right
clockwise	146	0	0	2	0	0	1
counterclockwise	0	147	1	0	0	0	0
left	0	0	144	0	0	0	0
nod head	0	7	0	166	0	0	0
m left	0	4	0	0	145	0	0
m right	5	0	0	4	0	140	0
right	0	0	2	0	0	0	141

**Table 4:** This table presents the same confusion matrix as in Table 3; however, each value is calculated relatively to the count of head gestures of each type.

	Clockwise	Counterclockwise	Left	Nod Head	M Left	M Right	Right	Error
clockwise	0.980	0.000	0.000	0.013	0.000	0.000	0.007	0.020
counterclockwise	0.000	0.993	0.007	0.000	0.000	0.000	0.000	0.007
left	0.000	0.000	1.000	0.000	0.000	0.000	0.000	0.000
nod head	0.000	0.040	0.000	0.960	0.000	0.000	0.000	0.040
m left	0.000	0.027	0.000	0.000	0.973	0.000	0.000	0.027
m right	0.034	0.000	0.000	0.026	0.000	0.940	0.000	0.060
right	0.000	0.000	0.014	0.000	0.000	0.000	0.986	0.014

## References

[1] Kim M., Choi S.H., Park K.B., Lee J.Y. (2019). User Interactions for Augmented Reality Smart Glasses: A Comparative Evaluation of Visual Contexts and Interaction Gestures. Appl. Sci..

[2] Kangas J., Rantala J., Akkil D., Isokoski P., Majaranta P., Raisamo R. (2017). Vibrotactile Stimulation of the Head Enables Faster Gaze Gestures. Int. J. Hum. Comput. Stud..

[3] Morales J., Akopian D. (2017). Physical activity recognition by smartphones, a survey. Biocybernetics Biomed. Eng..

[4] Farooq M., Sazonov E. (2018). Accelerometer-Based Detection of Food Intake in Free-Living Individuals. IEEE Sens. J..

[5] Ahuja K., Islam R., Parashar V., Dey K., Harrison C., Goel M. (2018). EyeSpyVR: Interactive Eye Sensing Using Off-the-Shelf, Smartphone-Based VR Headsets. Proc. ACM Interact. Mob. Wearable Ubiquitous Technol..

[6] Mavuş U., Sezer V. Head gesture recognition via dynamic time warping and threshold optimization. Proceedings of the 2017 IEEE Conference on Cognitive and Computational Aspects of Situation Management (CogSIMA).

[7] Yi S., Qin Z., Novak E., Yin Y., Li Q. GlassGesture: Exploring head gesture interface of smart glasses. Proceedings of the IEEE INFOCOM 2016—The 35th Annual IEEE International Conference on Computer Communications.

[8] Kelly D., Delannoy D., McDonald J., Markham C. Automatic recognition of head movement gestures in sign language sentences. Proceedings of the 4th China-Ireland Information and Communications Technologies Conference.

[9] Morimoto C., Yacoob Y., Davis L. Recognition of head gestures using hidden Markov models. Proceedings of the 13th International Conference on Pattern Recognition.

[10] Hasna O.L., Potolea R. Time series—A taxonomy based survey. Proceedings of the 2017 13th IEEE International Conference on Intelligent Computer Communication and Processing (ICCP).

[11] Shokoohi-Yekta M., Hu B., Jin H., Wang J., Keogh E. (2017). Generalizing DTW to the Multi-dimensional Case Requires an Adaptive Approach. Data Min. Knowl. Discov..

[12] Xue Y., Ju Z., Xiang K., Chen J., Liu H. (2019). Multimodal Human Hand Motion Sensing and Analysis—A Review. IEEE Trans. Cognitive Dev. Syst..

[13] Cheng H., Yang L., Liu Z. (2016). Survey on 3D Hand Gesture Recognition. IEEE Trans. Circuits Syst. Video Technol..

[14] Dalmazzo D., Ramírez R. (2019). Bowing Gestures Classification in Violin Performance: A Machine Learning Approach. Front. Psychol..

[15] Parnandi A., Uddin J., Nilsen D.M., Schambra H.M. (2019). The Pragmatic Classification of Upper Extremity Motion in Neurological Patients: A Primer. Front. Neurol..

[16] Huang J., Zhou W., Li H., Li W. (2018). Attention-Based 3D-CNNs for Large-Vocabulary Sign Language Recognition. IEEE Trans. Circuits Syst. Video Technol..

[17] Liu J., Li Y., Song S., Xing J., Lan C., Zeng W. (2019). Multi-Modality Multi-Task Recurrent Neural Network for Online Action Detection. IEEE Trans. Circuits Syst. Video Technol..

[18] ur Rehman M.Z., Waris M., Gilani S., Jochumsen M., Niazi I., Jamil M., Farina D., Kamavuako E. (2018). Multiday EMG-Based Classification of Hand Motions with Deep Learning Techniques. Sensors.

[19] Zhao H.Y., Wang Z., Qiu S., Xu F., Wang Z., Shen Y. (2019). Adaptive gait detection based on foot-mounted inertial sensors and multi-sensor fusion. Inf. Fusion.

[20] Switonski A., Josinski H., Wojciechowski K. (2019). Dynamic time warping in classification and selection of motion capture data. Multidimension. Syst. Signal Process..

[21] (2018). A Survey on Gait Recognition. ACM Comput. Surv..

[22] Berman S., Stern H. (2012). Sensors for Gesture Recognition Systems. IEEE Trans. Syst. Man Cybern. Part C Appl. Rev..

[23] Hachaj T., Ogiela M.R. Classification of Karate Kicks with Hidden Markov Models Classifier and Angle-Based Features. Proceedings of the 2018 11th International Congress on Image and Signal Processing, BioMedical Engineering and Informatics (CISP-BMEI).

[24] Billon R., Nédélec A., Tisseau J. Gesture Recognition in Flow Based on PCA and Using Multiagent System. Proceedings of the 2008 ACM Symposium on Virtual Reality Software and Technology.

[25] Bottino A., Simone M.D., Laurentini A. (2007). Recognizing Human Motion using Eigensequences. J. WSCG.

[26] Świtoński A., Mucha R., Danowski D., Mucha M., Polanski A., Cieslar G., Wojciechowski K., Sieron A. (2011). Diagnosis of the motion pathologies based on a reduced kinematical data of a gait. Przeglad Elektrotechniczny.

[27] Mantovani G., Ravaschio A., Piaggi P., Landi A. (2010). Fine classification of complex motion pattern in fencing. Procedia Eng..

[28] Choi W., Ono T., Hachimura K. Body Motion Analysis for Similarity Retrieval of Motion Data and Its Evaluation. Proceedings of the 2009 Fifth International Conference on Intelligent Information Hiding and Multimedia Signal Processing.

[29] Skurowski P., Pruszowski P., Pęszor D. (2016). Synchronization of Motion Sequences from Different Sources. AIP Conf. Proc..

[30] Hinkel-Lipsker J., Hahn M. (2018). Coordinative structuring of gait kinematics during adaptation to variable and asymmetric split-belt treadmill walking – A principal component analysis approach. Hum. Movement Sci..

[31] Yang Y., Zeng L., Leung H. Keyframe Extraction from Motion Capture Data for Visualization. Proceedings of the 2016 International Conference on Virtual Reality and Visualization (ICVRV).

[32] Lee M., Roan M., Smith B. (2009). An application of principal component analysis for lower body kinematics between loaded and unloaded walking. J. Biomech..

[33] Zago M., Pacifici I., Lovecchio N., Galli M., Federolf P., Sforza C. (2017). Multi-segmental movement patterns reflect juggling complexity and skill level. Hum. Movement Sci..

[34] Peng S. Motion Segmentation Using Central Distance Features and Low-Pass Filter. Proceedings of the 2010 International Conference on Computational Intelligence and Security.

[35] Fotiadou E., Nikolaidis N. (2014). Activity-based methods for person recognition in motion capture sequences. Pattern Recognit. Lett..

[36] Choi W., Li L., Sekiguchi H., Hachimura K. Recognition of gait motion by using data mining. Proceedings of the 2013 13th International Conference on Control, Automation and Systems (ICCAS 2013).

[37] Choi W., Sekiguchi H., Hachimura K. Analysis of Gait Motion by Using Motion Capture in the Japanese Traditional Performing Arts. Proceedings of the 2009 Fifth International Conference on Intelligent Information Hiding and Multimedia Signal Processing.

[38] Chalodhorn R., Rao R.P.N., Sigaud O., Peters J. (2010). Learning to Imitate Human Actions through Eigenposes. From Motor Learning to Interaction Learning in Robots.

[39] Das S.R., Wilson R.C., Lazarewicz M.T., Finkel L.H. (2006). Two-Stage PCA Extracts Spatiotemporal Features for Gait Recognition. J. Multimedia.

[40] Hachaj T., Piekarczyk M., Ogiela M.R. (2017). Human Actions Analysis: Templates Generation, Matching and Visualization Applied to Motion Capture of Highly-Skilled Karate Athletes. Sensors.

[41] Hachaj T., Choraś M., Choraś R.S. (2020). Head Motion—Based Robot’s Controlling System Using Virtual Reality Glasses. Image Processing and Communications.

[42] Field M., Pan Z., Stirling D., Naghdy F. (2011). Human motion capture sensors and analysis in robotics. Ind. Rob..

[43] Kim H., Gabbard J.L., Anon A.M., Misu T. (2018). Driver Behavior and Performance with Augmented Reality Pedestrian Collision Warning: An Outdoor User Study. IEEE Trans. Visual Comput. Graphics.

[44] Li G., Chung W. (2018). Combined EEG-Gyroscope-tDCS Brain Machine Interface System for Early Management of Driver Drowsiness. IEEE Trans. Hum. Mach. Syst..

[45] Bao H., Fang W., Guo B., Wang P., Ahram T., Falcão C. (2018). Real-Time Eye-Interaction System Developed with Eye Tracking Glasses and Motion Capture. Advances in Human Factors in Wearable Technologies and Game Design.

[46] Chui K.T., Alhalabi W., Liu R.W. (2019). Head motion coefficient-based algorithm for distracted driving detection. Data Technol. Appl..

[47] Zhang C., Wu X., Zheng X., Yu S. (2019). Driver Drowsiness Detection Using Multi-Channel Second Order Blind Identifications. IEEE Access.

[48] Karatas C., Liu L., Gruteser M., Howard R. Single-Sensor Motion and Orientation Tracking in a Moving Vehicle. Proceedings of the 2018 15th Annual IEEE International Conference on Sensing, Communication, and Networking (SECON).

[49] Zhao Y., Görne L., Yuen I.M., Cao D., Sullman M., Auger D.J., Lv C., Wang H., Matthias R., Skrypchuk L. (2017). An Orientation Sensor-Based Head Tracking System for Driver Behaviour Monitoring. Sensors.

[50] Kela J., Korpipää P., Mäntyjärvi J., Kallio S., Savino G., Jozzo L., Marca S.D. (2005). Accelerometer-based gesture control for a design environment. Pers. Ubiquitous Comput..

[51] LSM6DS3 iNEMO inertial module: always-on 3D accelerometer and 3D gyroscope. www.st.com/web/en/resource/technical/document/datasheet/DM00133076.pdf.

[52] Petitjean F., Ketterlin A., Gançarski P. (2011). A Global Averaging Method for Dynamic Time Warping, with Applications to Clustering. Pattern Recogn..

[53] Markley L., Cheng Y., Crassidis J., Oshman Y. (2007). Averaging Quaternions. J. Guidance Control Dyn..

[54] Breiman L. (1996). Bagging Predictors. Mach. Learn..

[55] Hachaj T. (2019). Improving Human Motion Classification by Applying Bagging and Symmetry to PCA-Based Features. Symmetry.

[56] Liu W., Sun J., Li W., Hu T., Wang P. (2019). Deep Learning on Point Clouds and Its Application: A Survey. Sensors.

[57] Chen Y., Zhou L., Bouguila N., Zhong B., Wu F., Lei Z., Du J., Li H. Semi-Convex Hull Tree: Fast Nearest Neighbor Queries for Large Scale Data on GPUs. Proceedings of the 2018 IEEE International Conference on Data Mining (ICDM).

[58] Li K., Malik J. Fast K-nearest Neighbour Search via Dynamic Continuous Indexing. Proceedings of the 33rd International Conference on International Conference on Machine Learning.

[59] Recht B., Ré C., Wright S.J., Niu F., Shawe-Taylor J., Zemel R.S., Bartlett P.L., Pereira F., Weinberger K.Q. (2011). Hogwild: A Lock-Free Approach to Parallelizing Stochastic Gradient Descent. Advances in Neural Information Processing Systems 24 (NIPS 2011).

